# The role of anti-Müllerian hormone: insights into ovarian reserve, primary ovarian insufficiency, and menopause prediction

**DOI:** 10.1007/s12020-025-04265-0

**Published:** 2025-05-23

**Authors:** Eleftheria Karaviti, Dimitra Karaviti, Eleni-Rafaela Kani, Efstathia Chatziandreou, Stavroula A. Paschou, Theodora Psaltopoulou, Sophia Kalantaridou, Irene Lambrinoudaki

**Affiliations:** 1 Endocrine Unit and Diabetes Centre, Department of Clinical Therapeutics, Alexandra Hospital, School of Medicine, National and Kapodistrian University of Athens, Athens, Greece; 2 Second Department of Obstetrics and Gynecology, Aretaieion University Hospital, School of Medicine, National and Kapodistrian University of Athens, Athens, Greece; 3 Third Department of Obstetrics and Gynecology, Attikon University Hospital, School of Medicine, National and Kapodistrian University of Athens, Athens, Greece

**Keywords:** Menopause, AMH, Anti-mullerian hormone, Primary ovarian insufficiency

## Abstract

This review highlights the role of Anti-Müllerian Hormone (AMH) in ovarian insufficiency and as a predictor of menopause. AMH, produced by granulosa cells in growing follicles, is a key marker of ovarian reserve, reflecting the remaining pool of viable follicles. In cases of primary ovarian insufficiency (POI), AMH levels are significantly reduced, aiding in diagnosis and distinguishing POI from other causes of amenorrhea. AMH levels below 8 pmol/L have shown high sensitivity (85%) and specificity (100%) for diagnosing POI in women with secondary oligomenorrhea. Regarding ovarian aging, AMH declines steadily with age, serving as a reliable predictor of menopausal timing. AMH levels are linked to menopausal symptoms, particularly vasomotor symptoms like hot flashes and their severity. However, its reliability for diagnosing menopause is inconsistent, especially in younger populations or when determining the exact onset. AMH levels can predict an earlier onset of menopause with limited sensitivity and specificity, particularly when using age-specific concentrations, as lower age-specific AMH levels are associated with an earlier menopause onset. Tracking AMH over time can improve the prediction of menopause. The accuracy of AMH measurements can be enhanced when considered alongside other hormonal markers or clinical symptoms. In polycystic ovary syndrome (PCOS), elevated AMH levels suggest a delayed onset of menopause, indicating an approximately two-year longer reproductive lifespan compared to women without PCOS (mean menopause age: 51.4 years in PCOS cases vs. 49.7 years in healthy controls). In endometriosis, AMH levels generally decline after surgery; however, they remain stable after chemotherapy, even years later, indicating that the decline in ovarian reserve may not be significantly affected.

## Introduction

AMH, or Müllerian inhibiting substance, is an essential glycoprotein with significant roles in both sexual differentiation and reproductive health. Although initially recognized for its role in male fetal development, in which it stimulates the regression of the Müllerian ducts, much emphasis has been placed on the broader implications of AMH, particularly in female reproductive physiology. In women, AMH secretion occurs through the granulosa cells of ovaries and plays an important role in folliculogenesis regulation. It acts, therefore, as a good indicator of ovarian reserve. Its activity extends both at gonadotropin-dependent and independent phases of follicle growth, contributing to a deeper understanding of fertility, ovarian function, and reproductive disorders such as polycystic ovary syndrome (PCOS). The purpose of this review is to discuss the multifunctional nature of AMH. It focuses on several of its mechanisms of action, its regulation, and the interplay existing between AMH, gonadotropins, and factors from the environment in reproductive health. Additionally, AMH is highly valued as an important biomarker of female reproductive aging in the context of menopause and the linked climacteric symptoms. Because of its close association with ovarian reserve, AMH is characterized by a gradual decline with woman age approaching menopause and reflects the gradual loss of ovarian follicles. In contrast to other hormones, such as FSH and estradiol, AMH remains stable throughout the menstrual cycle and hence is considered an efficient marker of the climacteric phase, or the transitional period to menopause. This period is very often accompanied by physical, psychological, and vasomotor symptoms that may seriously affect a woman’s quality of life. During the last years, a huge number of research works have been focused on investigating the predictive value of AMH in menopausal transition and its possible relation with climacteric symptom severity. Whereas some confirm that low levels of AMH are indicative of earlier menopause, others highlight the variability of AMH’s predictive accuracy, considering the differences between several health conditions such as POI, PCOS, and iatrogenic ovarian impairment due to medical treatments. This review summarizes the physiological functions and clinical relevance of AMH in fertility diagnosis, ovarian deficiency, and menopause, according to recent studies. It also further explores the potential value of AMH as both a predictor and a diagnostic tool for menopause and provides information on its clinical utility in health and well-being management in women during this life transition..

### Function of antimüllerian hormone

Anti-Müllerian hormone (AMH), also known as Müllerian inhibiting substance (MIS), is a glycoprotein and a member of the TGF-β family primarily produced by Sertoli cells in the fetal testis to promote the regression of Müllerian ducts, which would otherwise develop into the fallopian tubes, uterus, and proximal vagina, thus preventing the development of female internal genitalia [1, 2]. In females, AMH is mainly expressed by granulosa cells of growing follicles [3, 4]. AMH signals through two types of serine/threonine kinase receptors: the type II receptor, which is specific to AMH and widely expressed in various female reproductive organs, and the type I receptors. The type I receptors—ALK2/ACVR1, ALK3/BMPR1A, or ALK6/BMPR1B—as well as the SMAD1, SMAD5, or SMAD8 intracytoplasmic signaling proteins, are shared by AMH and bone morphogenetic proteins (BMPs). In addition to the SMAD signaling pathway, AMH can also activate the nuclear factor-κB (NF-κB) pathway in breast cancer cell lines [3, 4].

The regulation of AMH and its receptor (AMHR2) expression in the ovary is a complex process involving various transcription factors, cofactors, epigenetic mechanisms, hormones, and environmental factors.

The human AMH promoter features binding sites for several key transcription factors involved in sexual differentiation. In the fetal testis, Steroidogenic Factor 1 (SF1), an orphan nuclear receptor, is crucial for initiating AMH expression, working alongside the Sex-determining Region Y (SRY)–related HMG Box 9 (SOX9) in male sexual differentiation to stimulate Sertoli cell differentiation and AMH expression [5, 6]. GATA4 and Wilms Tumor 1 protein (WT1) also contribute to AMH regulation [7, 8]. In the fetal ovary, AMH expression is repressed by genetic cascades involving the forkhead-type transcription factor FOXL2 and the RSPO1/WNT/CTNNB1 pathway, which inhibit the male-specific differentiation program [9, 10]. However, during folliculogenesis, FOXL2 and WNT4 may induce AMH expression, with FOXL2 up-regulating AMH in granulosa cells and playing a key role in SF1-induced AMH regulation [11, 12]. SF1 and GATA4, also involved in testicular AMH expression, are up-regulated in preantral follicle granulosa cells and may affect ovarian AMH levels [7]. The AMHR2 promoter also contains sites for SF1 and GATA4, which activate it in heterologous cells [13, 14], and is influenced by WT1 and the β-catenin/CTNNB1 signaling pathway [15, 16]. Both AMH and AMHR2 expression are regulated by epigenetic mechanisms, such as long non-coding RNAs (lncRNAs), with H19lncRNA being crucial for reducing AMH expression and lncRNA-AMHR2 enhancing AMHR2 promoter activity in granulosa cells [17].

Gonadotropins impact AMH and AMHR2 expression, with FSH promoting AMH expression in various species. FSH and its second messenger cAMP up-regulate AMH in vitro, such as in human luteinized granulosa cells, but elevated estrogen levels often inhibit AMH expression, creating an inverse correlation between AMH and FSH during ovarian stimulation [18, 19]. Recombinant FSH can normalize AMH levels in isolated hypogonadotropic hypogonadism patients [20]. LH has a less clear effect on AMH, with in vitro studies showing no significant impact on human luteinized granulosa cells [21, 22].

Steroids, particularly estrogens, generally inhibit AMH and AMHR2 expression. Estradiol (E2) reduces AMH and AMHR2 mRNA levels and inversely correlates with AMH during folliculogenesis, with varying effects depending on follicle stage and estrogen receptor levels [23, 24]. Androgens, produced by the theca cells of follicles, have a complex role in AMH regulation, with mixed findings across studies. Some studies show no significant androgen effect on AMH, while others suggest a potential influence in conditions like polycystic ovary syndrome (PCOS) [25–27] BMPs stimulate AMH and AMHR2 expression, with BMP15, often with GDF9, enhancing AMH expression. BMP2, BMP4, and BMP6 also promote AMH and AMHR2 expression, with BMP4 activating AMH through SF1 [28–30], and BMP15 being essential for AMHR2 expression in granulosa cells [30, 31].

Metabolic and inflammatory factors also regulate AMH expression. Insulin up-regulates AMH mRNA levels, while leptin’s effects vary [32–34]. Inflammatory factors like VEGF and TNF-α can repress AMH expression [35, 36], and IGF and leptin influence AMHR2 expression [32, 37].

Environmental factors, including vitamin D, tobacco, and endocrine disruptors, may affect AMH and AMHR2 expression, though results are inconsistent [38–40], and psychological factors such as chronic stress can lead to abnormal AMH levels [41, 42]. Further research is needed to fully understand the roles of additional factors like inhibin B and IGF-1, as well as environmental and societal influences on AMH and AMHR2 expression. In vitro studies are essential to elucidate these regulators and the underlying molecular mechanisms.

Effects of AMH on Female Reproductive Organs

### The function of AMH

AMH secretion starts at the primary follicle stage [1, 2] and peaks at the stage when the follicle becomes selectable—pre-antral stage in mice and antral stage in humans. After this, AMH secretion from granulosa cells decreases as the follicles enter the FSH-dependent cyclic recruitment process [1]. AMH is believed to influence folliculogenesis in two distinct phases. First, it negatively regulates the initial recruitment of follicles in both mice and humans by inhibiting the transition from primordial to primary follicle [43, 44]. Second, it suppresses the FSH-dependent cyclical recruitment. AMH appears to be involved in follicular growth that is independent of gonadotropins.

Experimental in vitro studies on prepubescent mice lacking the AMH gene [45] further support this ongoing role of AMH. In vivo, AMH-deficient knockout mice (AMH -/-) exhibit a substantial increase in the number of growing follicles at all stages, including those beyond the pre-antral follicle stage, when compared to control mice [45]. Furthermore, adding AMH to cell cultures containing follicles from AMH -/- mice slowed follicular growth, even in the presence of FSH, indicating that AMH inhibits FSH-dependent proliferation of granulosa cells [46].

#### Gonadotropin-independent phase

In this phase, follicle growth occurs moderately with the involvement of FSH, but FSH is not essential. Instead, other stimulating factors such as androgens, kit-ligand, leukemia inhibitory factor (LIF), and growth differentiation factor-9 (GDF9) support basal follicle growth [47].

Androgens enhance FSH receptor (FSHR) expression in granulosa cells (GCs) through androgen receptors (AR), amplifying follicular growth independent of estrogen [48–51].

AMH plays a regulatory role by potentially inhibiting premature differentiation of GCs and reducing their sensitivity to FSH, thereby delaying follicle growth [46, 52]. In conditions such as polycystic ovary syndrome (PCOS), elevated AMH levels may contribute to slower follicle development [53].

**Fig. 1:**
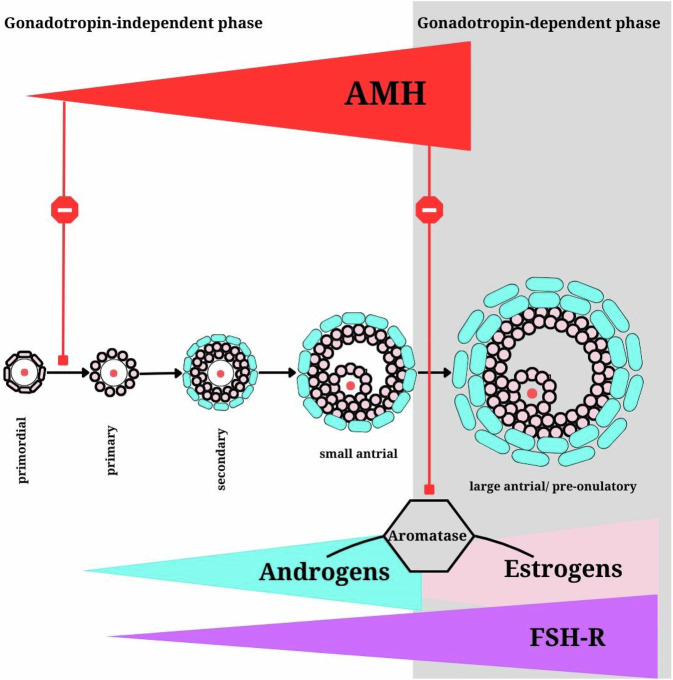
From the secondary stage onward, growing follicles secrete androgens. Androgens enhance the expression of FSH receptors (FSH-R), facilitating FSH-driven follicular growth and maturation. However, AMH acts as a protective mechanism, preventing premature follicle selection by FSH. It achieves this by inhibiting both the transition from primordial to primary follicles and the FSH-induced expression of aromatase, thereby blocking the conversion of androgens into estrogens

**Fig. 2:**
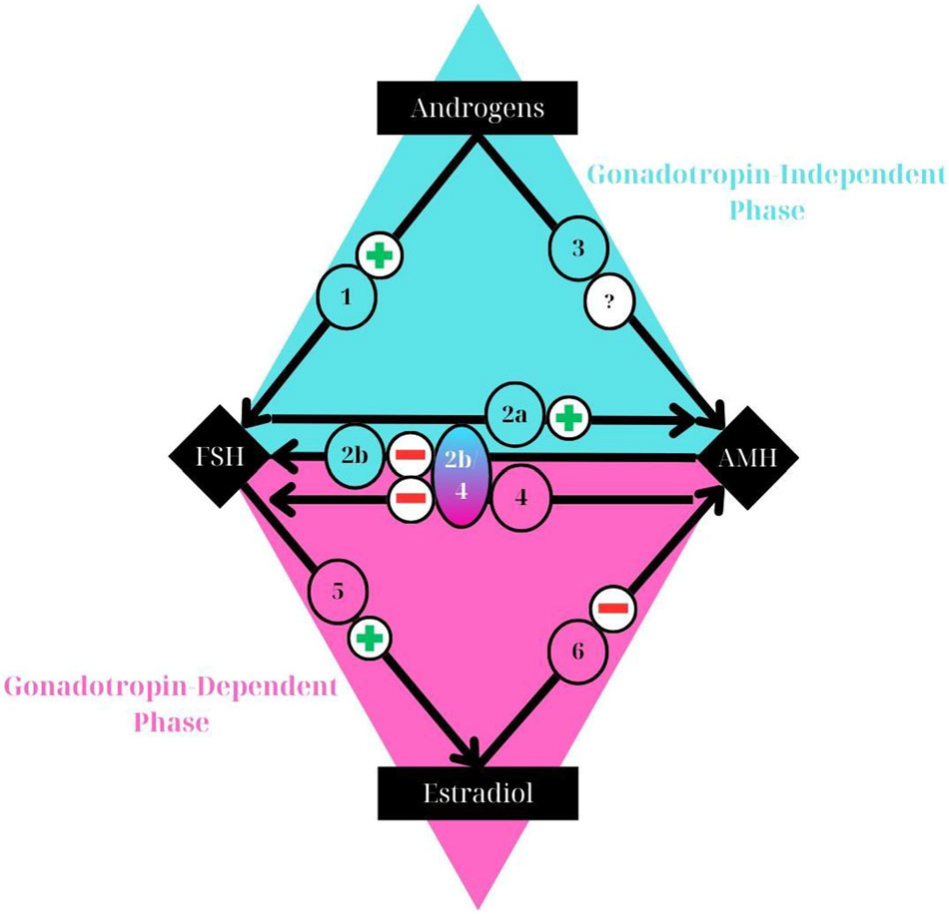
Relationships between androgens, FSH, and AMH during the gonadotropin-independent follicular growth phase (blue triangle), and between FSH, AMH, and estradiol during the gonadotropin-dependent follicular growth phase (pink triangle). The symbols “+”, “−“, and “?” represent positive, negative, or uncertain effects of one factor on another. In the gonadotropin-independent phase, FSH, whose receptors are enhanced by androgens (arrow 1), stimulates AMH production (arrow 2a) when estradiol is absent. AMH, in turn, inhibits FSH, thereby blocking the promotion of follicular growth (arrow 2b). The direct effect of androgens on AMH production remains uncertain (arrow 3). In the gonadotropin-dependent phase, AMH inhibits cell differentiation by blocking FSH function (arrow 4), specifically by inhibiting aromatase induction (arrow 5). Over time, the inhibitory effect of AMH on FSH decreases, allowing FSH to induce aromatase, which leads to estradiol synthesis. This estradiol further accelerates the decline of AMH secretion in large antral follicles (arrow 6)

The relationship between androgens and AMH in females remains unclear, with some studies indicating that androgens may increase AMH expression in PCOS, while others show conflicting results [54, 55] Figs. 1, 2.

#### Gonadotropin-dependent phase

During this phase, FSH becomes critical for GC differentiation and the synthesis of estradiol (E2) in antral follicles. As follicles mature, FSH drives these processes, leading to a diminished influence of androgens [56, 57].

The interplay between AMH and FSH is complex and inconsistent; early studies suggested FSH might reduce AMH levels, while others indicated FSH can stimulate AMH expression [58–60]. Additionally, AMH inhibits FSH-induced aromatase activity, which in turn limits E2 production [61, 62].

Estradiol has been shown to repress AMH expression, especially during follicle maturation. This inverse relationship facilitates the transition from androgen dominance to estrogen dominance as follicles develop [19, 63].

Finally, AMH influences follicular selection by making certain follicles more resistant to FSH, ensuring that only the most viable follicles are selected for ovulation while others undergo atresia [1, 64].

### Physiology of menopause

Menopause represents a new period in a woman’s life when reproductive ability is gradually declined and eventually lost due to complete exhaustion of ovarian follicles [65, 66]. Menopause consists of 3 phases; the perimenopause or climacteric phase, menopause and post-menopause. The menopausal transition is a gradual progression, characterized as the period from the onset of irregular menstrual cycles, up to the final end of menstruation, due to the depletion of oocytes, marking menopause [67, 68]. The mean time of menopause occurrence is the age of 51 years ranging from 46–52 years [66–69]. Menopause is diagnosed after 12 consecutive months without menstruation [70]. By the term ´early menopause´ is characterized the onset of menopausal transition in earlier age than 45 years and this condition is observed in approximately 10% of women [67]. By the endocrinological aspect, the climacteric phase is a condition of hormonal fluctuation and changes leading to climacteric symptoms such as vasomotor symptoms (hot flashes and sweating), sleep disorders, and psychosomatic symptoms [66]. During perimenopause, the intervals between each menstrual period are increased at more than 60 days [71]. Estrogen insufficiency (mainly estradiol (E2)) is a key marker of menopause and it is a result of ovarian follicular exhaustion [72, 73]. Another one sequel of diminished ovarian follicle reserve is the markedly elevated serum levels of follicle-stimulating hormone (FSH) [71]. Increased FSH bloodstream concentration causes negative feedback, leading, finally, to declination of inhibin-B levels [71]. Estradiol levels continue to get lower, while FSH levels progressively rise for approximately two years after the final menstrual cycle. Accompany to the changes of FSH and inhibin-B levels, AMH levels and antral follicle count (AFC) are very low [71]. As a sequel of fluctuation and changes in hormonal levels there is not provoked the appropriate interactions between the hypothalamus, pituitary gland and ovaries (HPO axis) and it results in absence of menstrual cycles [74].

### Antimüllerian hormone and climacteric symptoms

As there is a strong correlation among serum concentration of AMH and the number of viable follicles remaining and developing in the ovaries both the levels of AMH in bloodstream and the quantity of ovarian follicles, progressively, decline as women approach menopause [75, 76]. Levels of AMH remains stable during menstrual cycle in contrast to follicle-stimulating hormone (FSH) and estradiol levels [77]. Hence, AMH seems to be a valuable marker of reproductive aging and menopausal transition, known as climacteric phase. The transition to menopause is often linked to a range unfavorable outcomes [78]. AMH serum levels gradually decrease during the reproductive years, with a significant decline at menopause, eventually becoming undetectable shortly thereafter [79, 80]. This degression of AMH causes deterioration of ovarian function and as a sequel, leads to depression of estrogen levels, hormonal imbalances that trigger common menopausal symptoms. There have been published many studies in the literature that examine the potential role of AMH as an indicator of climacteric phase [81]. The results of all of these studies prove that reduced AMH serum concentration was correlated with earlier onset of menopause and the manifestation of climacteric symptoms [25, 82, 83]. There are several symptoms that accompany the onset of menopause and according to the Greene climacteric scale they are classified in some main categories which are vasomotor symptoms (VMS), physical and psychosomatic symptoms, sexual and phycological symptoms [84, 85]. More specifically, vasomotor symptoms include hot flashes and sweating (especially in the night), while headaches, feeling of dizziness, difficulties in breathing, tiredness, feeling of pressure in head or body, muscle and joint pain, numbing are some somatic symptoms. Also, the category of psychological symptoms includes rapid heartbeat, sleeping and concentrating difficulties, depression, irritability, anxiety and loss of interest [86]. These symptoms can persist for seven years for the majority of women, with 20% reporting VMS even for 15 years after the menopausal transition [74]. Other symptoms that are linked to the onset of climacteric phase are mood swings, cognitive changes, low libido, mastodynia, heavy menstrual bleeding perimenopausal and urogenital atrophy [71, 77, 87]. Chatziandreou et al. explored the relationship between AMH and the onset of climacteric symptoms in a study, using the data of 180 Greek premenopausal women revealing a negative association between AMH levels and the intensity of VMS (r-coefficient = −0.321, *p*-value = 0.014) in the premenopausal Greek population of women who have irregular menstrual cycles. However, it should be noted that this observation is only specific for the VMS and not for the psychological, somatic or sexual symptoms [84]. Dhanoya et al. reported that higher AMH serum levels were linked to a reduced possibility of experiencing hot flashes in the past two weeks [88]. Cameron et al. exported some results which showed that declined AMH levels after chemotherapy treatment were associated with a higher possibility of VMS and other psychosomatic manifestations [87]. Lastly, there are many studies in the literature which examine certain groups of patients and do not support the correlation between AMH concentration and climacteric symptoms [89]. Further research into the relationship between AMH and menopausal symptoms would be extremely important as it could lead to refine its clinical applications and improve our understanding of reproductive aging.

### AMH and menopause diagnosis

Only two studies examined undetectable AMH as a menopause marker (de Vet et al. [90]; Shin et al. [91]), with neither providing strong evidence for its use as a formal diagnostic tool. More specifically, Shin et al., who their study includes 144 women aged 20–59 year, reported that undetectable AMH had equivalent diagnostic accuracy to elevated FSH (where 22.3 mIU/ml was the optimal cut-off point). However, the diagnostic accuracy may have been overestimated, as the study sample included women with established and not recent menopause [91]. As a marker of ovarian aging, de Vet et al. found that the serum levels of AMH, which are gradually declined over time in young normo-ovulatory women, are associated with the number of antral follicles and age, with a weaker correlation to FSH levels [90]. A further analysis of 288 healthy women aged 40–55 years also found that age has a greater effect on AMH levels than menopausal stage [92]. Another study, the SWAN study, found that very low AMH levels could predict imminent menopause, but with lower accuracy in women under 48 years compared in women over 51 years. In contrast, a detectable but low AMH more reliably predicted that menopause was not imminent. Meanwhile, more studies are needed to determine whether the combination of AMH measurement with symptoms, or other biomarkers, will improve diagnostic accuracy for menopause [81, 93].

### AMH and prediction of menopause

Lower AMH levels were strongly associated with an increased risk of early menopause or the onset of menopause-related symptoms, independent of known risk factors [94]. According to a study, log-AMH levels are inversely associated with postmenopausal status in Greek women, even after adjusting for age. Women with AMH levels ≥ 0.012 ng/dL reached menopause later than those with AMH levels < 0.012 ng/dL. Additionally, the findings indicate that AMH levels are inversely associated with the severity of VMS, but not to the overall severity of climacteric symptoms or other categories of climacteric symptoms [84]. Also, assessment of age-specific AMH concentrations indicated that lower levels at younger ages were associated with earlier menopause than unadjusted AMH levels for age [95]. For instance, a study predicted that an AMH of 0.1 ng/dl at age 26 was linked to menopause at 36 years, but at age 44, it predicted menopause at 48 [96]. Models for menopause prediction using AMH concentrations associated with age, either directly [97, 98] or through age-specific centiles [99, 100], adding valuable context. This is important because AMH trajectories converge with the age as ovarian reserve decreased. [101, 102]. Two more studies suggested that continuously monitoring of the rate of AMH over time can improve the accuracy of predicting menopause onset. In this case, following AMH decrease can provide a more detailed picture, which is relying on a lot of measurements than on a single one [81, 95, 97].

Moreover, one cohort study found that using AMH values at ages 25 and 30 underestimated the risk of early menopause before age 45 [103]. In contrast, for women aged 35 to 48 years, both low baseline AMH and a rapid decrease were associated with a shorter time to menopause [104]. This contrast occurs because of the differences in methodology or participant age ranges between the two studies. While the time between measurements was similar in both studies, Penn Ovarian Aging Study (POAS) modeled the decrease of AMH to undetectable levels before menopause to explain the model’s superior performance [104]. The monitoring of undetectable AMH or further decline on already low levels indicates follicular depletion and impending menopause, typically seen with increasing age. Additionally, age-related changes in the ovaries involve increased fibrosis and stromal functional changes; these, too, might further affect follicle activation and the accuracy of AMH estimates. It might be that the elucidation of such interactions could lead to a better interpretation of AMH in terms of the prediction of menopause. However, multiple AMH measurements are not needed to increase predictive accuracy [105].

### The role of AMH in diagnosis and prediction of menopause in women with POI

The central role of AMH in primary ovarian insufficiency (POI) among women has been investigated by many studies in various contexts. A small number of studies observed, a progressive decrease in AMH levels in women, who experienced a decreased ovarian function due to POI, although it remained normal in many women until POI developed (approximately 6% of the POI population) [106–108]. Another study also reported that most women with undetectable or extremely low AMH levels ( ≤ 0.25 ng/ml (1.78 pmol/l)) compared to their age-mate control counterparts was diagnostic of POI, strengthening the meaning of AMH in the diagnosis of POI. [58, 109]. More specific, a longitudinal study reported that AMH levels under 8 pmol/l before age 36 are associated with 17% possible appearance of POI in 5 years [110].

Moreover, due to the diagnostic accuracy of AMH, it is much easier to differentially diagnose the causes of oligo/amenorrhea, especially PCOS, hypogonadotropic hypogonadism and hyperprolactinemia, where AMH levels are characteristically normal or high and to assessment family members who have a family history of POI. A study based on different POI phenotypes showed that AMH levels, although they were consistently low, were lower in women with primary amenorrhea than in those with secondary amenorrhea [107]. AMH levels are generally very low in women with POI, and a threshold of 8 pmol/l (1.12 ng/ml) has shown 85% sensitivity and 100% specificity in diagnosing POI in a cohort of women with secondary oligomenorrhea [108]. Another study found that women with AMH below this threshold were more likely to have a family history of POI [111]. However, the Doetinchem Cohort Study [77] showed a low discriminatory performance of AMH in predicting menopause in young women with family history of POI. Therefore, the general utility of AMH as a diagnostic marker of menopause in young women with family history of POI remains to be confirmed [112].

Additionally, AMH concentrations in women without POI tends to have strong correlation with follicle counts [113]. According to the study of Fanchin et al., which aim is to examine potential changes in serum anti-Müllerian hormone (AMH) levels during controlled ovarian hyperstimulation (COH), serum AMH levels gradually decrease during multiple follicular maturation cycles, likely reflecting the significant reduction in the number of small antral follicles due to COH, while confirming the minimal AMH expression in larger follicles [58]. According to the ovarian biopsies of some women who were diagnosed with POI, they have 15 or more follicles [58].

Also, the use of systemic hormonal contraception might affects AMH levels, which are lower in women using systemic hormonal contraceptives than in women not taking any contraceptive [107, 114]. In conclusion, although measuring serum AMH holds potential as part of an endocrine panel for assessing women with oligo/amenorrhea or perimenopausal symptoms, its use for predicting the exact age of spontaneous menopause in individuals remains unreliable and is not currently recommended [81]. While it is understandable that separate discussions are necessary, it is worth emphasizing that premature ovarian insufficiency, early menopause, and physiological menopause can, at least under certain conditions, be considered as a continuum in the expression of ovarian aging rather than completely distinct clinical entities. The progression from POI to early menopause and ultimately to natural menopause may share overlapping biological mechanisms, including follicular depletion, genetic predisposition, and environmental influences. Recognizing this continuum provides a more integrated perspective on ovarian aging and its implications for reproductive and endocrine health.

### The role of AMH in diagnosis and prediction of menopause in women with PCOS

PCOS is a common reproductive endocrine disorder, characterized by hyperandrogenism, polycystic ovaries, and anovulation, with unique implications for reproductive aging. Most women with prior anovulation become ovulatory by age 40. Studies suggest that menopause in women with PCOS is delayed by over four years compared to regularly ovulating women. Using AMH levels to predict menopause in PCOS patients indicates a reproductive lifespan extension of about two years. Elevated AMH levels in these women may reflect a larger follicle pool, contributing to a later menopause [115]. Minooee et al. found that serum concentration of AMH among PCOS participants (5.4 ng/ml) was significantly higher than in the controls (1.4 ng/ml). The estimated mean age at menopause was 51.4 years in PCOS cases and 49.7 years in healthy controls [116]. However, long-term studies connecting early AMH measurements to actual menopause age in PCOS patients are still lacking [115]. Beyond its role in predicting menopause, AMH is also considered a valuable biomarker for the diagnosis of PCOS. Women with PCOS typically exhibit AMH levels that are two to three times higher than those of women without PCOS [117]. Moreover, some studies suggest AMH thresholds higher than 3.8–5 ng/mL may improve diagnostic accuracy [118]. AMH has proven to be a valuable biomarker in the diagnosis of PCOS, providing significant advantages over traditional diagnostic methods. Its strong association with antral follicle count and its role in the pathophysiology of PCOS highlights its potential as both a diagnostic and prognostic indicator. AMH plays a significant role in the pathophysiology of PCOS by affecting both folliculogenesis and androgen production. AMH is produced by granulosa cells and its role in regulating follicular development is essential. High AMH levels in women with PCOS are associated with an increased number of small antral follicles potentially leading to follicular arrest and persistent anovulation. Some studies indicate that AMH may stimulate androgen production by ovarian theca cells, potentially worsening hyperandrogenism in PCOS patients [119, 120]. The strong correlation of AMH with antral follicle count and the pathophysiology of PCOS underscores its potential as both a diagnostic and prognostic tool.

### The role of AMH in diagnosis and prediction of menopause in women with endometriosis

Endometriosis, particularly ovarian endometriosis, raises concerns about fertility and timing of menopause. An ovarian endometrioma could potentially reduce ovarian reserve because of the destroy of the ovarian tissue. Streuli et al. reported no direct relationship between endometriosis or ovarian endometriomas and lower AMH levels [121]. In contrast, Uncu et al. found that women with endometriomas had lower AMH levels compared with controls before laparoscopic excision surgery [122]. A recent systematic review, which aim was to evaluate the effect of unilateral versus bilateral ovarian endometriomas on ovarian reserve biomarkers before and after cystectomy, found no differences in AMH between women with unilateral and bilateral endometriomas, challenging the idea that endometriomas impair ovarian reserve. Α significant decrease of AMH levels in serum was observed from 39.5–57.0%, from baseline to post-surgery. Surgical treatment is most consistently associated with long-term decline in ovarian reserve [77, 123]. Some research documents that ovarian endometriosis surgery impairs ovarian function, with several consequences, including a reduction in ovarian reserve highlighted by a drastic decrease in AMH serum levels. Considering the risk of further ovarian reserve depletion with repeated surgeries, surgical management for endometriosis should be carefully planned, especially for patients who want to conceive.

### AMH and iatrogenic ovarian reserve impairment

Iatrogenic ovarian reserve impairment affects women who have undergone treatments that may impact their dormant primordial follicle pool. In girls with cancer surviving childhood, the level of AMH is mostly a good marker of ovarian reserve following gonadotoxic chemotherapy [124]. AMH levels commonly undergo a great reduction shortly after chemotherapy, reflecting the acute effect on ovarian function. However, AMH levels typically experience a significant decline immediately following chemotherapy, reflecting the acute impact on ovarian function [125]. A longitudinal examination of childhood survivors of cancer who had measurable levels of AMH post-treatment documented their AMH decline rate to be on par with a control group, but this observation should not be misinterpreted as evidence of unchanged ovarian reserve [126]. Instead, it highlights the complexity of ovarian function recovery and the individual variability in reproductive aging post-treatment. Furthermore, the question is open whether AMH can accurately identify menopause age in this group, as correlation between menopause timing and the level of AMH is broad. Lower concentrations of AMH have been unexpectedly found in the girls with primary cancer, even before the first treatment was instituted, possibly secondary to defective repair mechanisms for DNA, which is also supposed to influence the time of menopause [127–129]. Given these complexities, assertions that AMH levels are stable after chemotherapy and that reduction in ovarian reserve is not significantly affected should be reassessed with caution in light of the multifactorial determinants of post-treatment ovarian function.

### Limitations- variations in measurement of ΑΜΗ

The quantitative results of AMH measurements can show variance. This can be ascribed to the lack of standardized calibration, utilization of different AMH assays, inconsistency about which isoform to measure, sample handling, storage conditions, and batch-to-batch variation. Automated assays have improved sensitivity, but the absence of calibration against an international standard leads to inter-assay bias [130] Manufacturers use proprietary calibrators, which result in inconsistent standard curves and measurements [131, 132] While some measurands have international standards, AMH standardization is challenging due to considerable effort, costs, and resources that make the process difficult and slow [133]. In 2014, WHO approved the development of an AMH standard, and recombinant AMH SS-581 demonstrated stability [131]. However, a follow-up study using recombinant AMH 16/190 showed high assay variability due to differing calibrators and non-human protein matrices. WHO assigned a mass of 489 ng/ampoule to 16/190, but its commutability was unsatisfactory, preventing it from becoming a full international standard [134, 135]. It was instead designated as a reference reagent to aid manufacturers in reassessing calibrator values and improving harmonization [134].AMH measurement is complicated by isoforms with varying biological activity, detected differently by assays [136]. Variability in antibody selection impacts the detection of active and inactive AMH forms, potentially leading to misclassification of clinical conditions [137, 138]. Comparative studies highlight the influence of antibody selection and assay design on sensitivity and accuracy [139]. Interferences such as heterophile antibodies and biotin also affect AMH measurement, with some assays more susceptible to these effects [119, 140–143]. Automated assays have enhanced sensitivity and detection limits, improving clinical utility [144, 145]. However, the overall accuracy depends on random and systematic errors, with automated methods showing lower variability and better precision than manual ELISA tests [133, 135, 146]. The need for an international reference standard is critical, as discrepancies across assays emphasize the importance of traceability and harmonization for reliable clinical decision-making (Table 1).

**Table 1:** Anti-Müllerian hormone levels: measurements and correlations across various studies

Study	Publication year	Type of study	Number of participants	AMH assays	Outcomes
de Vet et al. [90]	2002	Longitudinal observational study and perspective	41 normo-ovulatory premenopausal women and 13 healthy postmenopausal women	Active MIS/AMH ELISA, DSL	AMH decreased over time (median 2.1 μg/l at Visit 1 versus 1.3 μg/l at Visit 2). AFC, FSH, and inhibin B did not change. AMH correlated with age, AFC, and FSH, but not with inhibin B. All postmenopausal women had undetectable AMH
Van Rooij et al. [148]	2004	Prospective longitudinal cohort	81 normal women between 25 and 46 years, with follow-up at an average interval of 4 years	EIA AMH/MIS, Immunotech	Lower AMH levels were linked to the onset of cycle irregularity. AMH, AFC, and age showed the highest predictive accuracy for irregular cycles. After adjusting for age, only AMH and inhibin B remained significantly associated with cycle irregularity.
Shin et al. [91]	2008	Cross-sectional	111 Healthy, ovulatory women (20–49 years) and 33 postmenopausal women	Access AMH, Beckman Coulter	AMH was lower in older women and undetectable in 20 of 33 postmenopausal women. The diagnostic accuracy of AMH for menopausal status was similar to an FSH > 22.3 mIU/ml and LH > 8.7 mIU/ml
J van Disseldorp et al. [149]	2008	prospective cross-sectional cohort	Prospect-European Prospective Investigation into Cancer and Nutrition study (Prospect-EPIC). Healthy, regularly cycling women, plus a subgroup of women aged 58 years and older who had undergone natural menopause	Active MIS/AMH ELISA, DSL; EIA AMH/MIS, Immunotech Conversion Factor	The observed similarity between predicted and actual menopause age supports the link between AMH levels and menopause onset. AMH can more accurately reflect a woman’s reproductive age compared to relying on chronological age alone.
Sowers et al. [150]	2008	Prospective longitudinal cohort	Michigan Bone Health and Metabolism Study. Subgroup of 50 women with regular cycle and FSH	Active MIS/AMH ELISA, DSL	Longitudinal AMH levels dropped to near the limit of detection approximately five years before the final menstrual period (FMP). Baseline AMH levels showed a significant association with the age at which FMP occurred.
Tehrani et al. [96]	2009	Prospective longitudinal cohort	Tehran Lipid and Glucose Study (TLGS). 60 of the 147 women entered the menopause during 6 years of follow-up. Mean age at baseline was 44.8 year	Active MIS/AMH ELISA, DSL	Among women in late reproductive age with AMH levels above 0.39 ng/mL, only 1 in 10 will reach menopause within the next six years. A single AMH measurement is a strong predictor for menopause onset.
Broer et al. [100]	2011	prospective longitudinal cohorts	Three cohorts, 257 women. Cohort 1: 172 women aged 25– 46 years who had a regular menstrual cycle; Cohort 2: 90 women aged 18– 46 years, two ovaries, no adnexal surgery in the past, and a regular menstrual cycle; Cohort 3: 40 normo-ovulatory women	Cohort 1: Active MIS/AMH ELISA, DSL Cohort 2 and 3: EIA AMH/MIS, Immunotech Conversion factor	AMH was linked to age at menopause. In an age-adjusted model, the hazard ratio for time to menopause was 0.092 (95% CI: 0.025–0.340) for each 0.89 ng/mL change in AMH levels.
Tehrani et al. [151]	2011	Prospective longitudinal cohort	Tehran Lipid and Glucose Study (TLGS). 266 women with three repeat measurements of AMH at around 3-year intervals. 63 women entered menopause over 6 years of follow-up. Mean age at baseline was 37.6 years	Active MIS/AMH ELISA, DSL	AMH levels were linked to age at menopause. A single baseline AMH measurement provided the best model accuracy, with no improvement from repeated measurements. The agreement between predicted and actual menopause age decreased for women at the younger and older age extremes.
Freeman et al. [106]	2012a	Prospective longitudinal cohort	Penn Ovarian Aging Study. Subgroup of 293 late reproductive age premenopausal women who had detectable AMH at baseline and at least one further AMH measurement. Mean age: 40.93 years at baseline. 14 years of follow-up. 146 became postmenopausal	AMH Gen II ELISA, Beckman Coulter	Rate of change of AMH was strongly associated with TTM and offered better accuracy when combined with baseline AMH levels, compared to relying on a single AMH measurement or age alone.
Freeman et al. [97]	2012b	Prospective longitudinal cohort	Penn Ovarian Aging Study. Subgroup of 401 premenopausal women with a baseline AMH measurement. Mean age at baseline: 41.5 years. 198 women reached natural menopause over 14 years of follow-up	AMH Gen II ELISA, Beckman Coulter	AMH effectively predicted time to menopause (TTM), with age improving accuracy. Women with baseline AMH < 0.20 ng/ml had a median TTM of 5.99 years (ages 45–48) and 9.94 years (ages 35–39); higher AMH levels showed longer TTM.
Tehrani et al. [152]	2013	Prospective longitudinal cohort	Tehran Lipid and Glucose Study (TLGS). 277 women reached the menopause over mean of 10 years of follow-up. Mean age at baseline: 36.7 years	AMH Gen II ELISA, Beckman Coulter	AMH at baseline was associated with age at menopause and improved an age alone model. The median difference between actual and predicted menopause age was 0.5 years, with prediction accuracy of 92% compared to 84% for age alone.
Iino et al. [153]	2013	Prospective longitudinal cohort	Subgroup of 44 women ( > 40 years old) from 595 women in Japan. Annual AMH measurements for 6 years. 29 women experienced menopause during the study	AMH Gen II ELISA, Beckman Coulter	Longitudinal reductions in AMH across the perimenopause; AMH may be a marker for defining menopausal transition
Ramezani, Tehrani et al. [98]	2014	Prospective longitudinal cohort	Scheffer, van Rooij, de Vet (SRV) cohort: 158 of 257 women at baseline followed up 11 years later; 48 became menopausal during follow-up. Tehran Lipid and Glucose Study (TLGS): 266 women were randomly selected from 1265 women; 63 became menopausal during follow-up	SRV: Active MIS/AMH ELISA, DSL; TLGS: AMH Gen II ELISA, Beckman Coulter Conversion factor	External validation of cohort-specific models for predicting menopause age based on AMH demonstrated strong alignment between the predicted risk and the actual age of menopause in both models.
Nair et al. [154]	2015	Prospective longitudinal cohort	Coronary Artery Risk Development in Young Adults (CARDIA) Women’s Study. Median age at baseline was 42 years, with 9 years of follow-up. 207 women reached natural menopause during follow-up	Ultra-Sensitive AMH/MIS ELISA, Ansh Labs	AMH is independently associated with age at menopause, showing the strongest predictive ability for identifying women at risk within three years. None of the women with AMH levels exceeding 2.0 ng/dL (20 pg/mL) experienced menopause within six years.
Dollemann et al. [155]	2015	Large perspective cohort study	Doetinchem Cohort Study. Assessment of menopause at 5 and 10 years of follow-up. 169 became postmenopausal at 5 years and 527 at 10 years of follow-up. Mean age at baseline: 40.8 years	AMH Gen II ELISA, Beckman Coulter	The model with AMH (including also age, BMI, years of smoking and menstrual cycle status) increased menopause risk prediction within 10 years in perimenopausal women by 3 and 14% in younger women.
De Kat et al. [77]	2016	Prospective longitudinal cohort	Doetinchem Cohort Study. Assessment of menopause at baseline, 5, 10, 15 and 20 years of follow-up. Repeat AMH measurements at 5-year intervals. In total, 1882 became menopausal. Mean age at baseline was 40 ± 10 years	picoAMH ELISA, Ansh Labs	Age-specific AMH was associated with the onset of menopause. AMH levels showed consistency across individual trajectories, with an ICC of 0.87. Higher AMH at 20 years before the final menstrual period (FMP) indicated a slower decline between 20 and 15 years prior. However, in the last five years before the FMP, higher AMH levels were linked to a faster decline.
Ramezani Tehrani et al. [156]	2016	Prospective longitudinal cohort	Tehran Lipid and Glucose Study (TLGS). 277 women reached menopause over a median of 9.8 years of follow-up	AMH Gen II ELISA, Beckman Coulter	Using age-specific AMH percentiles in a flexible parametric survival model enhanced the prediction of menopause. The discrepancy between the actual age and the predicted age at menopause was 1.9 years.
Gohari et al. [95]	2016	Prospective longitudinal cohort	Tehran Lipid and Glucose Study (TLGS). 63 of the 266 women entered menopause during an average of 6.5 years of follow-up, with AMH measured every 3 years. Mean age at baseline: 37.55 years	Active MIS/AMH ELISA, DSL	Rate of decline of AMH (AMH trajectory) using 2 or 3 measurements improved baseline AMH prediction of age of menopause
Depmann et al. [99]	2016	Pooled prospective longitudinal cohort study	265 women were at baseline between 1992 and 2001, 216 followed up between 2008–2010 or 2012–2013, and 155 were available for analyses. Mean age at baseline: 36 years. 81 women were postmenopausal, with a mean follow-up of 14 years	Active MIS/AMH ELISA, DSL EIA AMH/MIS, Immunotech	AMH predicts age at menopause, though its accuracy declines with increasing age, with broad prediction intervals and limitations in predicting extreme menopause ages.
Kim et al. [136]	2017	Prospective longitudinal cohort	Coronary Artery Risk Development in Young Adults (CARDIA) Women’s Study. 30 years of follow-up; mean age: 43 years	Ultra-Sensitive AMH/MIS ELISA, Ansh Labs	AMH improved age-based predictions of menopause within five years. Levels above 2.0 ng/dl indicated a low likelihood of menopause, while AMH below the limit of detection was linked to over 60% chance of menopause in women aged 45 and older.
Whitcomb et al. [157]	2018	Nested case-control study	Nested case-control subgroup (n = 820) of premenopausal women (n = 108,811) from the prospective longitudinal Nurses’ Health Study 2 (n = 116,429). Follow-up every 4 years. Mean age of sample: 33.8–34.2 years across the groups	picoAMH ELISA, Ansh Labs	Lower mean AMH was associated with a higher risk of early menopause
Bertone-Johnson et al. [96]	2018	Nested case-control study	Nurses’ Health Study 2. Women reporting natural menopause between sample collection and age 45, n = 327. Control group who experienced menopause after age 45, n = 491. Mean age in all groups: 40 years	picoAMH ELISA, Ansh Labs	AMH levels were significantly lower in women with early menopause (0.40 ng/ml) than controls (1.9 ng/ml; *P* < 0.001). F or each 0.10 ng/ml decrease, there was a 14% higher risk of early menopause.
de Kat et al. [103]	2019	Prospective longitudinal cohort	Doetinchem Cohort Study. Assessment of menopause at baseline, 5, 10, 15 and 20 years of follow-up. Repeat AMH measurements at 5-year intervals (367–736 women with 1–5 AMH measurements). In total, 1298 women became postmenopausal	picoAMH ELISA, Ansh Labs	In women over the age of 25 years, inclusion of repeat AMH measurements/AMH trajectory did not improve prediction of age of menopause or early menopause
Ramezani Tehrani et al. [158]	2020	Prospective longitudinal cohort	Tehran Lipid and Glucose Study (TLGS). 529 women reached menopause over a median follow-up period of 14 years. Mean age at baseline: 36 6 7.1 years	AMH Gen II ELISA, Beckman Coulter	Multiple AMH measurements could improve prediction of age at menopause; it will be useful in identifying women at risk of early menopause.
Soares et al. [83]	2020	Prospective longitudinal cohort	Avon Longitudinal Study of Parents and Children (ALSPAC). Four clinics over 5–6 years. 1608 women with 4037 observations over 5–6 years. At baseline, 281 were premenopausal, 493 perimenopausal and 547 postmenopausal.	Elecsys, AMH Plus, Roche Diagnostics	AMH decreased markedly before menopause and remained low subsequently. Women with earlier age at menopause had the highest levels of AMH about 4 years before the FMP, and the decline in AMH was slightly later than in those with menopause at on average 50 years
Finkelstein et al. [93]	2020	Prospective longitudinal cohort	The Study of Women’s Health Across the Nation (SWAN). Annual follow-up where possible until menopause (1537 women with 7407 blood samples) Mean age: 47.5 years	MenoCheck® picoAMH ELISA, Ansh Labs	AMH predicted the FMP within 12–36 months for late-reproductive aged women. Women with AMH < 10 pg/ml had a 51–79% chance of reaching menopause within 12 months, while those with AMH > 100 pg/ml had a 90–97% chance of not reaching menopause.
Ramezani Tehrani et al. [159]	2021	Prospective longitudinal cohort	Tehran Lipid and Glucose Study (TLGS). 522 women reached menopause over a median follow-up of 13 years. AMH levels were measured every 6 years. Mean age at baseline: 36 years	AMH Gen II ELISA, Beckman Coulter	A time-dependent Cox model demonstrated that each unit increase in AMH levels reduced the risk of menopause by 87%. The Cox proportional hazards model improved menopause age prediction by 3%. Multiple AMH measurements provided better individual risk predictions for physiological menopause than single measurements.
Zhang et al. [92]	2021	Cross-sectional	Women were categorized into three menopausal stages: late reproductive (n = 169), menopausal transition (n = 63), and early postmenopausal (n = 56). Median age was 48 years	Access AMH, Beckman Coulter	Both age and menopausal stage were linked to AMH levels, with age having a stronger influence than menopausal stage.
Chantziandreou et al. [84]	2023	Prospective longitudinal cohort	180 women (group A, 96 women of late reproductive stage/early perimenopause; group B, 84 women in late perimenopause)	Elecsys AMH Plus	Women with AMH levels ≥ 0.012 ng/dL experienced menopause later than those with AMH < 0.012 ng/dL. Additionally, AMI values were inversely related to the severity of vasomotor symptoms (VMS), but not to the overall severity of climacteric symptoms or other symptom groups.

## Conclusion

Anti-Müllerian hormone (AMH), produced by Sertoli and granulosa cells, plays a role in sexual differentiation and folliculogenesis [3, 4]. It prevents Müllerian duct development in males and regulates follicle recruitment in females [1, 2]. AMH expression is influenced by factors such as gonadotropins, steroids, BMPs, metabolism, and the environment [5, 37]. AMH can be a key biomarker for predicting menopausal age and assessing ovarian reserve [81, 90, 91]. AMH levels decline significantly with age, making it an essential indicator of ovarian function and reproductive health [76, 147]. Findings reveal that lower AMH concentrations are associated with an increased risk of early menopause, highlighting its potential for guiding clinical decisions regarding fertility and reproductive health management [85, 100]. AMH levels indicate ovarian reserve, decline with age, and become undetectable at menopause [80]. AMH levels are associated with menopausal symptoms, particularly vasomotor symptoms such as hot flashes [84, 88, 89]as well as their intensity [84]. Nonetheless, its reliability for diagnosing menopause varies, especially among younger individuals or when pinpointing the precise onset [95, 96]. In primary ovarian insufficiency, low AMH may help in diagnosis, but its accuracy is affected by factors like contraceptive use [107, 108, 114]. AMH levels can predict earlier menopause, especially with age-specific concentrations [95, 96], though monitoring over time improves accuracy. In conditions like polycystic ovary syndrome (PCOS), high AMH suggests delayed menopause [115, 116], while in endometriosis, AMH often declines post-surgery [77, 122, 123]. AMH also helps assess ovarian reserve in cancer survivors post-chemotherapy, though predicting menopausal age remains difficult [124–126]. The effectiveness of AMH improves when combined with other hormonal markers or clinical signs. Overall, more research is needed to refine its clinical application for predicting menopause and understanding reproductive aging.

## Data Availability

No datasets were generated or analysed during the current study.
